# Development and validation of a successful aging prediction model for older adults in China based on health ecology theory

**DOI:** 10.3389/fpubh.2025.1595540

**Published:** 2025-10-13

**Authors:** Zhucheng Zhang, Chenxi Peng, Zhuo Li, Jiaqiang Li, Yan Li, Yuhang Pan, Ruihong Liu, Xiangdong Chen

**Affiliations:** ^1^Department of General Practice, Health Science Center, Shenzhen University, Shenzhen, China; ^2^Department of Family Medicine, The University of Hong Kong-Shenzhen Hospital, Shenzhen, China; ^3^Department of Biomedical Engineering, Health Science Center, Shenzhen University, Shenzhen, China; ^4^Department of Otolaryngology-Head and Neck Surgery, Shenzhen University General Hospital, Shenzhen, China

**Keywords:** successful aging, prediction model, health ecology theory, machine learning, CHARLS

## Abstract

**Background and aim:**

Accelerated aging poses significant physical, psychological, and social health challenge to Chinese. Successful aging (SA) serves as a proactive approach to population aging, reflecting individual health status and quality of life, thereby enhancing the capacity for healthy living among the older adults. However, the complexity of SA measurement methods often hinders its application in community healthcare. Currently, there is a dearth of prediction model tailored for the older adults in community. This study aimed to develop and validate a prediction model for SA in Chinese community older adults.

**Methods:**

Data were derived from the fifth wave of the China Health and Retirement Longitudinal Study (CHARLS), targeting community-dwelling older adults individuals over 60. Employing health ecology theory, we comprehensively utilized variables from community health records. The Shapley Additive exPlanation (SHAP) method identified key variables contributing to outcome prediction. An extreme gradient boosting machine learning method was used to construct the prediction model for SA in Chinese community older adults. The final model was obtained through hyperparameter adjustment via 8-fold cross-validation. The model’s performance was evaluated using area under the receiver operating characteristic curves (AUROC), discriminant slope, calibration curves, decision curves, SHAP-based risk factor analysis, and comparison with other methods to assess differentiation, calibration, interpretability, and clinical utility.

**Results:**

The model incorporated variables available from community health records. SHAP indicated a robust importance ranking of variable features, with the most frequent top 16 features aligning with clinical practice, ensuring good interpretability and extensibility of the resulting prediction model. We used six machine learning methods to construct the prediction model. Among them, the extreme gradient boosting model demonstrated an AUROC of 0.78, a discrimination slope of 0.140, and a Brier score of 0.124. The proposed model is superior to other methods, and has outstanding discriminability and consistency. Decision curve analysis (DCA) indicated a higher clinical utility compared to other models.

**Conclusion:**

We proposed a prediction model for SA in Chinese community older adults based on health ecology theory and machine learning, which demonstrate excellent prediction performance, interpretability, and extensibility. The prediction model can be applied to community older population health management, promoting SA within community older adults.

## 1 Introduction

In the 21st century, the global demographic landscape is witnessing a pivotal transformation, particularly in the form of accelerated population aging. China, with the world’s largest older population, is facing this challenge. By the end of 2022, the number of older individuals aged 60 and above in China soared to 280,04 million, constituting 19.8% of the total population, a figure that not only surpasses previous records but also indicates an escalating trend (1, 2). This demographic shift exerts great pressure on the nation’s social security and healthcare infrastructures (3). It is important to note that community-based primary healthcare providers, as the sentinels of public health, play a crucial role in swiftly assessing the physical and mental well-being of the older population (4, 5).

The concept of Successful Aging (SA) is initially introduced by Havighurst in 1961 and later refined by Rowe and Kahn in 1987. It serves as a pivotal metric for evaluating the aging status of individuals and populations (6, 7). SA incorporates low levels of disease and disability, preserved cognitive and physical functionality, and active social engagement (8). As the older population grows, the ability to maintain good health and social participation becomes increasingly significant. Assessing the prevalence of SA within community-dwelling older populations is essential for proactively addressing the aging demography (9). The factors influencing SA are complex, as highlighted by Bronfenbrenner’s health ecology theory, which underscores the intricate interplay of multiple environmental levels in shaping human health (10). This theory posits that the health status of community-dwelling older adults is not an isolated phenomenon but is embedded within a health ecosystem that spans from micro to macro levels. At present, the World Health Organization’s Active Ageing model emphasizes health, participation and safety as the pillars of aging, which is consistent with our multi-dimensional approach to successful aging (physical, psychological, cognitive and social dimensions) (11, 12). Furthermore, Baltes’ SOC model, which focuses on adaptive strategies for aging, can clarify from another perspective how our research can predict and intervene in successful aging through measurable health and lifestyle factors, complementing this model (13).

Despite the burgeoning integration of SA and machine learning to evaluate the older population, many studies are constrained by ambiguous theoretical frameworks and the constraints of sample data source (14–16). There are limitations in the development of SA models with broad applicability, high differentiation, and significant clinical benefits. This study aims to leverage large, representative cross-sectional data from China to explore predictive factors of SA suitable for the older population in Chinese communities. By constructing a SA prediction model with strong interpretability, ease of application, and potential for widespread promotion in community settings, we aim to provide a convenient instrument for community assessment of the older adults. This instrument will facilitate targeted intervention measures and enhance the health and well-being of the older population in communities.

## 2 Materials and methods

### 2.1 Data sources and sample

A cross-sectional study design was employed in our research, utilizing data sourced from the fifth wave of the China Longitudinal Study of Health and Retirement (CHARLS) which is conducted in 2020. The CHARLS constitutes a nationally representative longitudinal investigation into aging, which has followed participants biennially since its baseline survey in 2011 (17). This comprehensive survey has collected an extensive array of detailed information pertaining to the health status, familial structures, health behaviors, income, expenditures, and policy on older adults. The CHARLS surveys (including the 2020 wave) have been approved by the Peking University Institutional Review Board for Biomedical Research, with ethics approval number IRB00001052-11015. The specific details can be found in previous studies (18).

In our study, we followed to the definitions established by the United Nations and the World Health Organization (WHO), defined individuals aged 60 years and above as older adults (19, 20). Additionally, we utilized data from the CHARLS questionnaire, which includes inquiries about the respondents’ residential status to ascertain whether they reside within the community.

The original data from CHARLS2020 included a total of 19,395 respondents. Consequently, we excluded participants who were below the age of 60, residing in the community, as well as datasets that were deemed incomplete, characterized by more than 70% missing data for critical variables.

The final set of variables in our analysis was derived through imputation using the “MICE” package within R Studio 4.1.2. We employed the “missForest” algorithm, a multivariate iterative random forest method, which was iterated five times with 100 estimators per iteration. This approach was selected to generate imputed datasets that minimize variance relative to the original dataset, ensuring the robustness and reliability of our subsequent analyses.

After the data cleaning process, we retained 4,324 eligible samples for inclusion in the data analysis. Among these, 614 individuals (14.20%) were identified as successful aging.

### 2.2 Outcome variable: successful aging

Aging is a multifaceted process that affects physical, mental, and social functioning. Rowe and Kahn have proposed a conceptual model of aging that describes aging as “normal” and “successful” (6, 8). Based on recent studies, specific measurements of the 5 criteria including “No major disease,” “No disability,” “No depression,” “High cognitive function” and “Active social engagement” (21–23). The specific details of potential predictors in this research are presented in Table 1. The outcome variable in this study was binary, which means that irrespective of whether community older population is successful aging or normal aging.

**Table 1:** Definition and measurement of successful aging.

Successful aging models^*^	Define	Measures
No major disease	No major chronic diseases	No chronic diseases: cancer, chronic lung disease, heart disease and stroke.
No disability	No restrictions in ADL	No difficulty in washing, dressing, toileting, carrying, feeding, controlling urination and defecation.
No depression	No depressive symptoms	CESD-10 score <10.
High cognitive function	Use TICS to get a median or higher score	A set of cognitive tests with scores ranging from 0 to 30. This includes immediate and delayed recall of 10 words, subtracting 7 words from 100 in a row (up to 5 times), drawing, answering the day of the week, year, month, day, and season.
Active social engagement	Get involved in social activities	Participate in “volunteer or charity work,” or “provide assistance to family, friends, or neighbors,” or “participate in sports, social, or other types of clubs,” or “interact with friends,” or “participate in community related organizations,” at least once a month.

^*^Rowe and Kahn’s model of successful aging. ADL, Activity of Daily Living; CESD-10, The Center for Epidemiological Studies Depression-10; TICS, The Telephone Interview for Cognitive Status.

### 2.3 Predictors and feature selection

Although the quantity of features plays a crucial role in model training, it is important to recognize that augmenting the number of features also escalates the level of difficulty and associated costs. Feature selection can be used to reduce the number of eliminate irrelevant or redundant features, thereby simplifying the final model and enhancing its efficiency (24).

**Figure 1:**
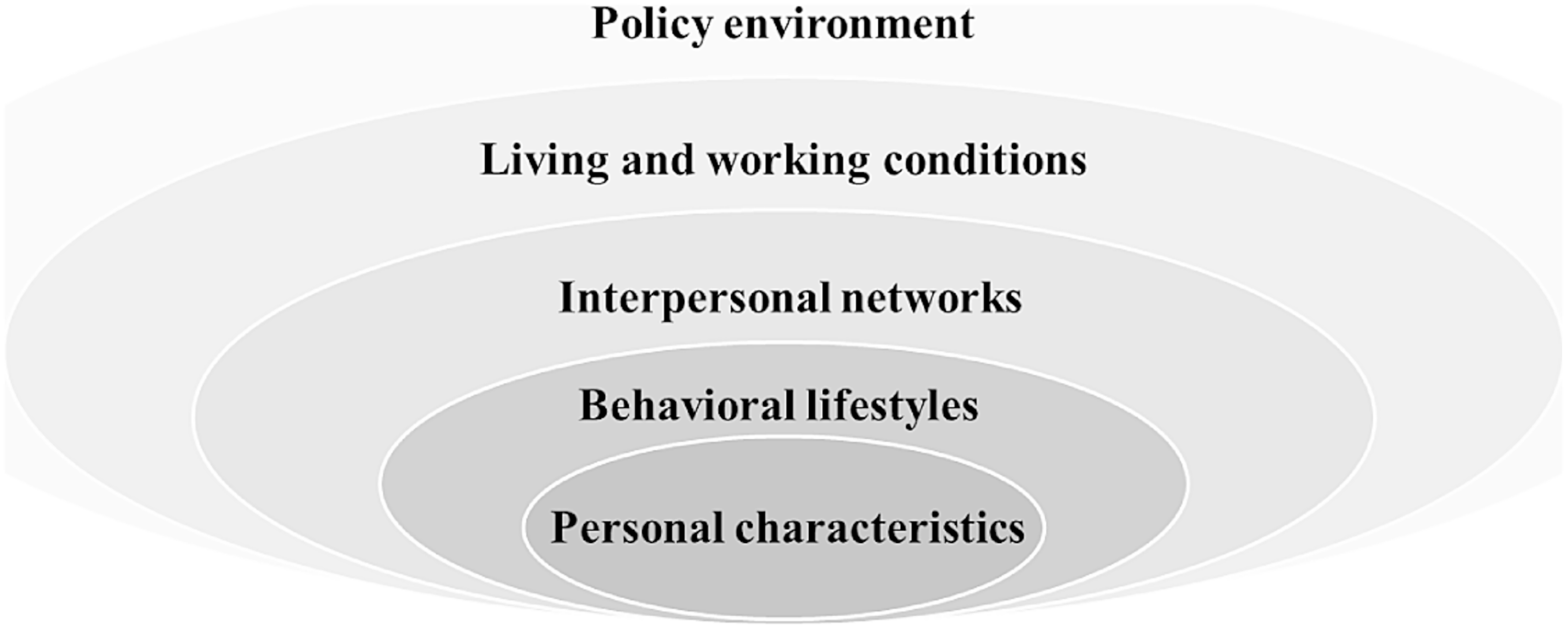
Model of health ecology theory.

Based on the health ecology theory and utilizing the health records of community residents that are employed by primary health care providers in China, we conducted a systematic review of pertinent studies to identify potential factors influencing successful aging in Figure 1 (25–28).

Subsequently, we performed feature extraction and utilized the Shapley Additive explanation SHAP to compute the top 30 most significant features for the model based on their experimental result. To determine the feature factors included in the model training, we observed the model’s AUC performance and employed a reclassification method while considering various feature sets. After consulting with experts specializing in primary care, nursing, and gerontology, we finally selected 16 predictors that are indicative of potential SA within Chinese communities. The potential predictors of SA include five dimensions as outlined by the health ecology theory: personal characteristics, behavior and lifestyle, interpersonal network, living and working conditions, and policy environment. The specific details of potential predictors are presented in Table 2.

**Table 2:** Predictive factors about SA.

Dimensions^*^	Variable name	Variable type	Concept and assignment
Personal characteristics	Age	Continuous variable	Age was calculated by time of interview and birth time.
	Gender	Categorical variable	Male = 1; female = 2.
	Self-rated health	Categorical variable	Good = 1; general = 2; bad = 3.
	Life satisfaction	Categorical variable	Very satisfied = 1; satisfied = 2; very dissatisfied = 3.
	Hypertension	Categorical variable	Yes = 1; no = 0.
	Dyslipidemia	Categorical variable	Yes = 1; no = 0. (dyslipidemia including high or low lipids)
	Diabetes or elevated blood sugar	Categorical variable	Yes = 1; no = 0. (diabetes or elevated blood sugar including impaired glucose tolerance and elevated fasting blood sugar)
	Malignant tumours	Categorical variable	Yes = 1; no = 0. (malignant tumours such as cancer excluding mild skin cancer)
	Chronic lung diseases	Categorical variable	Yes = 1; no = 0. (chronic lung diseases such as chronic bronchitis or emphysema, cor pulmonale, excluding tumours or cancers)
	Liver diseases	Categorical variable	Yes = 1; no = 0. (liver diseases excluding fatty liver, tumour or cancer)
	Heart disease	Categorical variable	Yes = 1; no = 0. (heart disease such as myocardial infarction, coronary heart disease, angina, congestive heart failure and other heart diseases)
	Stroke	Categorical variable	Yes = 1; no = 0.
	Kidney diseases	Categorical variable	Yes = 1; no = 0. (kidney diseases excluding tumors or cancers)
	Digestive diseases	Categorical variable	Yes = 1; no = 0. (diseases of the stomach or digestive system excluding tumours or cancers)
	Emotional and mental problems	Categorical variable	Yes = 1; no = 0.
	Diseases related to memory	Categorical variable	Yes = 1; no = 0. (diseases related to memory such as Alzheimer’s disease, brain atrophy)
	Parkinson’s Disease	Categorical variable	Yes = 1; no = 0.
	Arthritis or rheumatism	Categorical variable	Yes = 1; no = 0.
	Asthma	Categorical variable	Yes = 1; no = 0.
Behavioral lifestyles	Smoke	Categorical variable	Yes = 1; no = 0.
	Drink	Categorical variable	Yes = 1; no = 0.
	Night sleep time	Categorical variable	The duration of respondents’ sleep at night. 6–8 h = 1; < 6 h = 2; ≥ 8 h = 3.
	Physical activity	Categorical variable	The respondents’ exercise in the past month. Yes = 1; no = 0.
Interpersonal networks	Marital status	Categorical variable	Have spouse = 1; no spouse = 0.
	Satisfaction with child	Categorical variable	Very satisfied = 1; satisfied = 2; very dissatisfied = 3.
	Individual income	Categorical variable	All types of income for respondents in a single year. According to the quartile, it is divided into high = 1, medium high = 2, medium low = 3 and low = 4.
	Living alone	Categorical variable	Yes = 1; no = 0.
Living and working conditions	Living areas	Categorical variable	Eastern region = 1; central region = 2; western region = 3; northeastern region = 4.
	Residence	Categorical variable	Urban = 1; rural = 2.
	Education level	Categorical variable	Below primary school = 1; primary school = 2; middle school = 3; high school = 4; high school above = 5.
Policy environment	Medical insurance	Categorical variable	Have urban employee medical insurance = 1; government medical insurance = 2; private medical insurance = 3; urban and rural resident medical insurance = 4; other medical insurance = 5; none = 0.
	Pension	Categorical variable	Have = 1; none = 0.

*According to the health ecology theory, we classify the SA-related factors involved in the model in five dimensions. Detailed questionnaire details can be found in previous studies.

### 2.4 Model development

**Figure 2:**
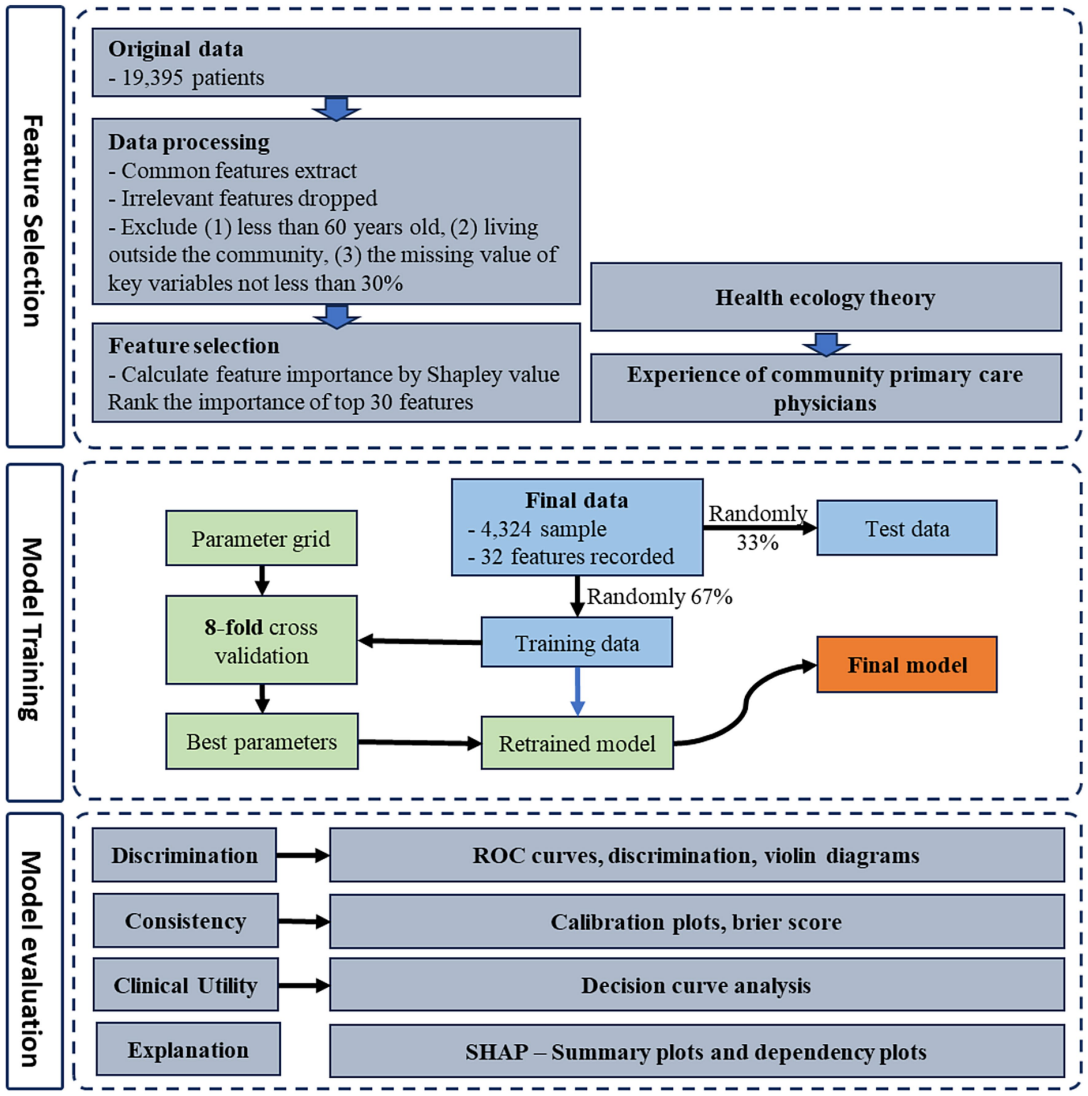
Technical roadmap for model development.

The research endeavor consists of a three-phased approach to model development: (1) construction of machine learning model, (2) model evaluation, and (3) model interpretation. The technical roadmap outlining this research methodology is depicted in Figure 2.

Prior to model construction, 67% of the samples were designated for the training set. The remaining 33% were allocated to the validation set.

In this study, we employed the scikit-learn Python library in conjunction with the XGBoost package for model construction and hyperparameter fine-tuning. To ensure consistent and comparable data, we applied a linear function normalization technique, specifically mapping the original data to the range [0,1]. This approach achieved proportional scaling, effectively mitigating any potential dimensional biases among the data features.

We applied six machine learning methods comprise (1) Logistic Regression (LR), (2) Random Forest (RF), (3) Light Gradient Boosting Machine (LGBM), (4) Gradient Boosting Decision Tree (GBDT), (5) K-Nearest Neighbors (KNN), (6) Extreme Gradient Boosting (XGBoost). These algorithms were compared to assess their performance in predicting SA among older individuals in the community.

#### 2.4.1 Model construction

##### 2.4.1.1 Logistic regression

Logistic Regression (LR), a type of general linear model, assumes that the outcomes adhere to a Bernoulli distribution, parameterized by p, which represents the likelihood of a positive outcome. In our study the positive outcome is the likelihood of SA among community-dwelling older adults. It is noteworthy that logistic regression imposes stringent requirements on the ratio of features to samples, necessitating a balance that ensures model generalizability across diverse populations. The parameters utilized in our study for logistic regression are set to their default values (29).

##### 2.4.1.2 Random forest

Random Forest (RF), an ensemble method based on the Bagging principle, constructs multiple decision trees to enhance classification performance. Each tree draws a random subset of samples and features. The final classification is determined by a majority vote among the trees. The study used 1,000 trees with default settings to evaluate the model’s ability to predict successful aging. The RF algorithm’s effectiveness is attributed to its bagging technique, which reduces variance and improves accuracy by aggregating predictions (30, 31).

##### 2.4.1.3 Light gradient boosting machine

Light Gradient Boosting Machine (LGBM) is a gradient boosting framework that leverages tree-based learning algorithms. It highlights two key techniques: Gradient-based One-Side Sampling (GOSS), which improves efficiency by focusing on data with substantial gradients during training, and Exclusive Feature Bundling (EFB), which simplifies the feature space by bundling exclusive features. These methods are crucial for enhancing the model’s ability to handle large-scale data and maintain accuracy (32).

##### 2.4.1.4 Gradient boosting decision tree

Gradient Boosting Decision Tree (GBDT) begins with a single decision tree and iteratively adds trees to correct the errors of previous ones. The model uses a gradient-based approach to maximize loss reduction at each split point. Regularization techniques are employed to prevent overfitting, and the model’s complexity is managed through parameters that control the number and depth of tree leaves. The predictions from individual trees are aggregated for a final prediction, leveraging the ensemble’s collective wisdom to reduce variance and improve accuracy. Hyperparameter tuning, using grid search cross-validation, is crucial for achieving optimal GBT model performance by balancing bias and variance (33).

##### 2.4.1.5 K-nearest neighbors

K-Nearest Neighbors (KNN) is a supervised learning technique used for classification tasks. The KNN algorithm determines the class of an unlabeled data point by finding the ‘K’ nearest labeled neighbors in the feature space and then assigning the class label based on the majority vote of these neighbors. The process hinges on three key aspects: a set of labeled training data, a distance metric to measure the proximity between data points, and the selection of the ‘K’ value. This method is particularly useful in biomedicine for tasks such as pattern recognition and classification, where understanding the proximity of data points can provide insights into complex relationships within the data (34).

##### 2.4.1.6 Extreme gradient boosting

Extreme gradient boosting (XGBoost) is a gradient boosting framework known for its efficiency and scalability in machine learning. XGBoost was first proposed in 2011, optimizes prediction models by reducing the loss function through gradient descent. It stands out for its ability to integrate multiple weak models into a high-performance model, a process that enhances prediction accuracy and generalizes well across different datasets. The algorithm’s regularization features also help in preventing overfitting (35).

To enhance the classification performance of our XGBoost model, we implemented an 80% cross-validation strategy for hyperparameter optimization. This process focused on fine-tuning key hyperparameters. Through an iterative approach, we identified the most effective parameter combinations, which were subsequently integrated into our refined model.

#### 2.4.2 Model evaluation

We had a rigorous evaluation of the proposed models, utilizing a suite of metrics to assess performance. The metrics encompass accuracy, precision, sensitivity, specificity, and the F1-measure. The formulas for calculating these metrics are delineated in Table 1. Additionally, we incorporated the area under the receiver operating characteristic curve (AUC-ROC) as a pivotal metric, quantifying the model’s ability to distinguish between different classes. The eightfold cross-validation approach was employed to ascertain the robustness of our algorithms.

To enhance the evaluative rigor of our model’s predictive capabilities, we incorporated decision curve analysis (DCA) (36). This method allowed us to scrutinize the model’s discriminatory power and diagnostic precision across a spectrum of subgroups, thereby enabling a nuanced comparison and targeted optimization of performance metrics. In parallel, we plotted a calibration curve to meticulously align predicted probabilities with actual outcomes. This visual representation is pivotal for a thorough evaluation of our model’s consistency and accuracy, ensuring that our predictive analytics not only perform well in theory but also align with empirical data in practice.

#### 2.4.3 Model interpretation

The SHAP method serves as a technique for explaining individual predictions and providing global interpretation (37). By calculating the contribution of each feature, it indicates the model’s decision-making process, ultimately yielding meaningful interpretation results. The SHAP summary plot and dependence plot offer visualization that illustrate how diverse features impact prediction outcomes. In this study, the SHAP algorithm was employed to indicate the importance of features within the prediction model. SHAP values were computed to delineate the relationship between input factors and the output, specifically revealing the contribution of various feature factors to the prediction of successful aging among community-dwelling older adults.

### 2.5 Model development

Descriptive statistical analysis was conducted for all variables in the study. Continuous variables were summarized using the median with interquartile range (IQR) or the mean with standard deviation (SD), depending on their distribution. Categorical variables were described by their proportion within each category.

Kruskal-Walli’s rank sum test and Chi-square test were used to compare the continuity variables of non-normal distribution and categorical variables, respectively. *p* < 0.05 was considered statistically significant. In this study, Python scripting language (v3.9.13) and R (v4.3.1) were used for analysis.

## 3 Results

### 3.1 Characteristics of the sample

Following the data processing phase of our retrospective study, a total of 4,324 medical records were amassed, comprising 614 individuals with SA and 3,710 without SA. The demographic distribution included 2,207 males (51.00%) and 2,207 females (49.00%), with a median age of the participants being 67.6 years, as delineated by interquartile ranges A–B. The chi-square test was employed to ascertain the key factors significantly associated with SA, with the outcomes detailed in Table 3. Subsequently, variables that demonstrated a *p*-value of less than 0.05 in the univariant regression analysis were identified and are also presented in Table 3.

**Table 3:** Characteristics of the study population in the training set.

Variable	All (*N* = 4,324)	Normal aging (*N* = 3,710)	Successful aging (*N* = 614)	*p*-value
Age	67.6 ± 5.87	67.9 ± 5.98	66.4 ± 4.98	**<0.001**
Gender				0.096
Male	2,207 (51.0%)	1874 (50.5%)	333 (54.2%)	
Female	2,117 (49.0%)	1836 (49.5%)	281 (45.8%)	
Self-rated health				**<0.001**
Good	1,120 (25.9%)	864 (23.3%)	256 (41.7%)	
General	2,240 (51.8%)	1911 (51.5%)	329 (53.6%)	
Bad	964 (22.3%)	935 (25.2%)	29 (4.72%)	
Life satisfaction				**<0.001**
Very satisfied	1,699 (39.3%)	1,442 (38.9%)	257 (41.9%)	
Satisfied	2,268 (52.5%)	1914 (51.6%)	354 (57.7%)	
Very dissatisfied	357 (8.26%)	354 (9.54%)	3 (0.49%)	
Hypertension				**0.030**
Yes	324 (11.5%)	288 (12.1%)	36 (8.33%)	
No	2,489 (88.5%)	2093 (87.9%)	396 (91.7%)	
Dyslipidemia				**0.014**
Yes	346 (9.53%)	314 (10.0%)	32 (6.43%)	
No	3,286 (90.5%)	2,820 (90.0%)	466 (93.6%)	
Diabetes or elevated blood sugar				**<0.001**
Yes	301 (7.18%)	301 (8.42%)	0 (0.00%)	
No	3,889 (92.8%)	3,275 (91.6%)	614 (100%)	
Malignant tumours				**0.002**
Yes	63 (1.46%)	63 (1.70%)	0 (0.00%)	
No	4,247 (98.5%)	3,633 (98.3%)	614 (100%)	
Chronic lung diseases				**<0.001**
Yes	286 (6.85%)	286 (8.02%)	0 (0.00%)	
No	3,892 (93.2%)	3,278 (92.0%)	614 (100%)	
Liver diseases				0.223
Yes	97 (2.34%)	88 (2.47%)	9 (1.55%)	
No	4,040 (97.7%)	3,468 (97.5%)	572 (98.5%)	
Heart disease				**<0.001**
Yes	380 (9.31%)	380 (11.0%)	0 (0.00%)	
No	3,702 (90.7%)	3,088 (89.0%)	614 (100%)	
Stroke				**<0.001**
Yes	161 (3.79%)	161 (4.43%)	0 (0.00%)	
No	4,089 (96.2%)	3,475 (95.6%)	614 (100%)	
Kidney diseases				**0.002**
Yes	163 (4.05%)	154 (4.46%)	9 (1.57%)	
No	3,863 (96.0%)	3,298 (95.5%)	565 (98.4%)	
Digestive diseases				0.066
Yes	219 (6.75%)	196 (7.11%)	23 (4.72%)	
No	3,024 (93.2%)	2,560 (92.9%)	464 (95.3%)	
Emotional and mental problems				0.074
Yes	37 (0.87%)	36 (0.99%)	1 (0.16%)	
No	4,220 (99.1%)	3,612 (99.0%)	608 (99.8%)	
Diseases related to memory				**<0.001**
Yes	173 (4.00%)	165 (4.45%)	8 (1.30%)	
No	4,151 (96.0%)	3,545 (95.6%)	606 (98.7%)	
Parkinson’s Disease				**0.027**
Yes	48 (1.11%)	47 (1.27%)	1 (0.16%)	
No	4,275 (98.9%)	3,662 (98.7%)	613 (99.8%)	
Arthritis or rheumatism				0.089
Yes	257 (9.39%)	224 (9.84%)	33 (7.17%)	
No	2,480 (90.6%)	2053 (90.2%)	427 (92.8%)	
Asthma				**0.029**
Yes	67 (1.59%)	64 (1.78%)	3 (0.49%)	
No	4,135 (98.4%)	3,526 (98.2%)	609 (99.5%)	
Smoke				**0.019**
Still smoking	1,152 (26.6%)	965 (26.0%)	187 (30.5%)	
Quit	598 (13.8%)	505 (13.6%)	93 (15.1%)	
Never	2,574 (59.5%)	2,240 (60.4%)	334 (54.4%)	
Drink				**<0.001**
Drink more than once a month	1,187 (27.5%)	958 (25.8%)	229 (37.3%)	
Drink less than once a month	375 (8.67%)	310 (8.36%)	65 (10.6%)	
Never	2,762 (63.9%)	2,442 (65.8%)	320 (52.1%)	
Night sleep time				**<0.001**
6–8 h	734 (17.0%)	584 (15.7%)	150 (24.4%)	
< 6 h	2,599 (60.1%)	2,270 (61.2%)	329 (53.6%)	
≥ 8 h	991 (22.9%)	856 (23.1%)	135 (22.0%)	
Physical activity				**<0.001**
Yes	369 (8.53%)	348 (9.38%)	21 (3.42%)	
No	3,955 (91.5%)	3,362 (90.6%)	593 (96.6%)	
Marital status				**<0.001**
Have spouse	3,312 (76.6%)	2,803 (75.6%)	509 (82.9%)	
No spouse	1,012 (23.4%)	907 (24.4%)	105 (17.1%)	
Satisfaction with child				**<0.001**
Very satisfied	2,341 (54.1%)	2011 (54.2%)	330 (53.7%)	
Satisfied	1786 (41.3%)	1,508 (40.6%)	278 (45.3%)	
Very dissatisfied	197 (4.56%)	191 (5.15%)	6 (0.98%)	
Individual income				**<0.001**
High	1,082 (25.0%)	1,006 (27.1%)	76 (12.4%)	
Medium high	1,069 (24.7%)	953 (25.7%)	116 (18.9%)	
Medium low	1,082 (25.0%)	933 (25.1%)	149 (24.3%)	
Low	1,091 (25.2%)	818 (22.0%)	273 (44.5%)	
Living alone				0.170
Yes	3,435 (79.4%)	2,934 (79.1%)	501 (81.6%)	
No	889 (20.6%)	776 (20.9%)	113 (18.4%)	
Living areas				**<0.001**
Eastern region	1,335 (30.9%)	1,105 (29.8%)	230 (37.5%)	
Central region	1,240 (28.7%)	1,044 (28.1%)	196 (31.9%)	
Western region	1,527 (35.3%)	1,367 (36.8%)	160 (26.1%)	
Northeastern region	222 (5.13%)	194 (5.23%)	28 (4.56%)	
Residence				**<0.001**
Urban	1,424 (32.9%)	1,110 (29.9%)	314 (51.1%)	
Rural	2,900 (67.1%)	2,600 (70.1%)	300 (48.9%)	
Education				**<0.001**
Below primary school	1,178 (27.2%)	1,107 (29.8%)	71 (11.6%)	
Primary school	998 (23.1%)	869 (23.4%)	129 (21.0%)	
Middle school	946 (21.9%)	803 (21.6%)	143 (23.3%)	
High school	757 (17.5%)	607 (16.4%)	150 (24.4%)	
High school above	445 (10.3%)	324 (8.73%)	121 (19.7%)	
Medical insurance				**<0.001**
None	169 (3.91%)	158 (4.26%)	11 (1.79%)	
Urban employee medical insurance = 1	637 (14.7%)	465 (12.5%)	172 (28.0%)	
Government medical insurance = 2	416 (9.62%)	369 (9.95%)	47 (7.65%)	
Private medical insurance = 3	170 (3.93%)	125 (3.37%)	45 (7.33%)	
Urban and rural resident medical insurance = 4	2,814 (65.1%)	2,507 (67.6%)	307 (50.0%)	
Other medical insurance = 5	118 (2.73%)	86 (2.32%)	32 (5.21%)	
Pension				**0.017**
Have	3,847 (89.0%)	3,283 (88.5%)	564 (91.9%)	
None	477 (11.0%)	427 (11.5%)	50 (8.14%)	

The continuous variable of normal distribution is expressed as mean ± standard deviation, and the categorical variable is expressed as sample size (ratio %). *p*<0.05 are bolded.

### 3.2 Feature selection for SA

**Figure 3:**
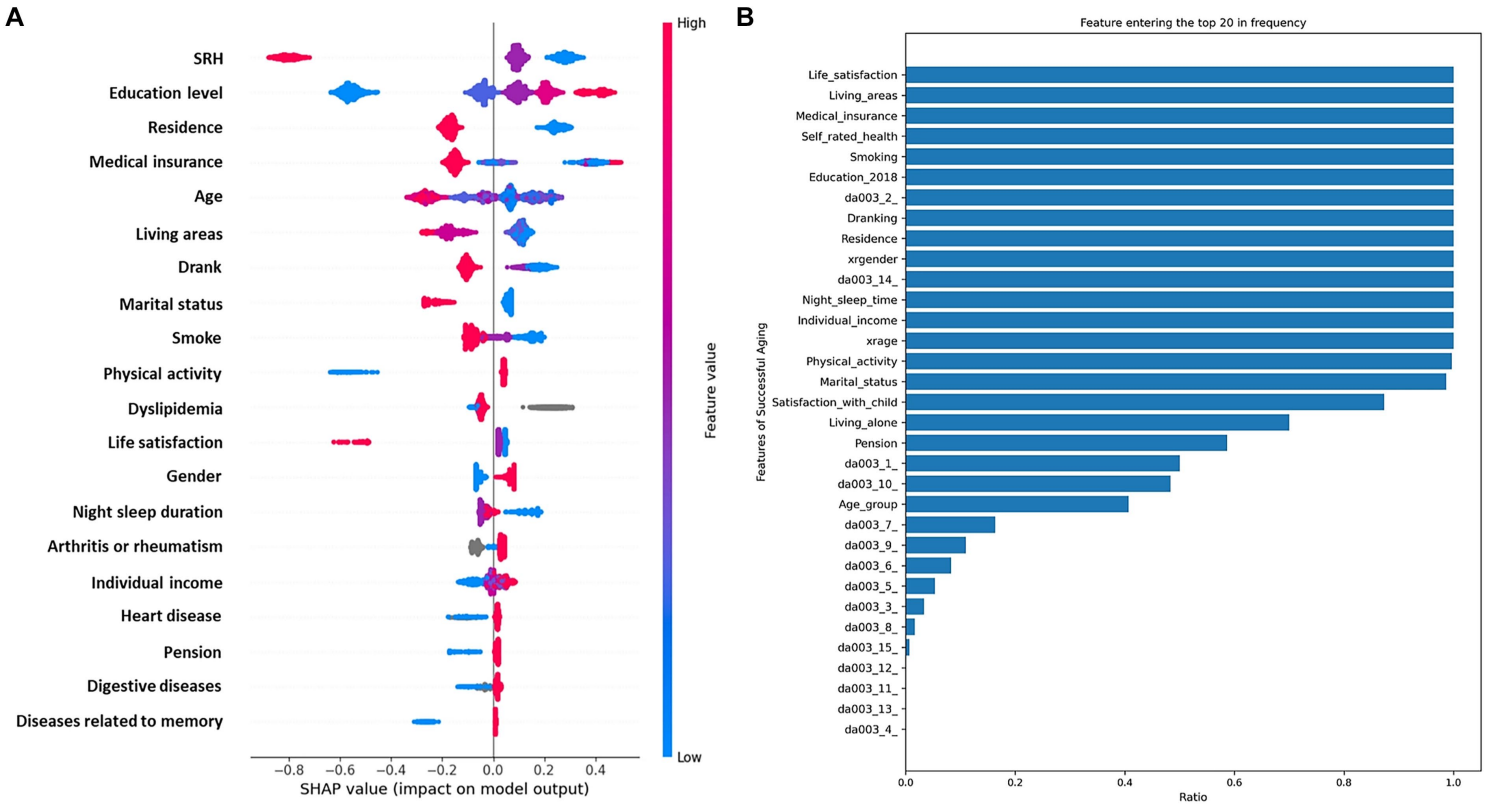
Integrated analysis of feature importance and impact distributions. **(A)**, The prominence of top 30 features based on SHAP importance rankings. **(B)**, The distribution of the impacts of each feature on the model output.

In our study, patients were randomly allocated to the training and test sets in a 2: 1 ratio to ensure a robust evaluation framework. The training set encompassed 32 distinct features, which were subjected to calculate SHAP values to quantify their individual contributions to the model’s predictive accuracy. Each feature’s contribution was ranked, and this ranking process was iterated 300 times to ascertain the consistency and reliability of the results. We then tallied the frequency with which each feature appeared in the top 30 across these iterations. The resulting feature rankings, which are depicted in Figure 3A, reveal the most influential variables in our model (due to space limitations, only the top 30 features are displayed here). We also elucidate the distributional impact of each feature’s influence on the model output in Figure 3B. Prominent among these were life satisfaction, living area, medical insurance, self-rated health (SRH), smoke, education level, dyslipidemia, drank, residence, gender, night sleep duration, individual income, age, physical activity, and marital status, all of which demonstrate significant importance across all assessments.

### 3.3 Performance comparison of different models

**Figure 4:**
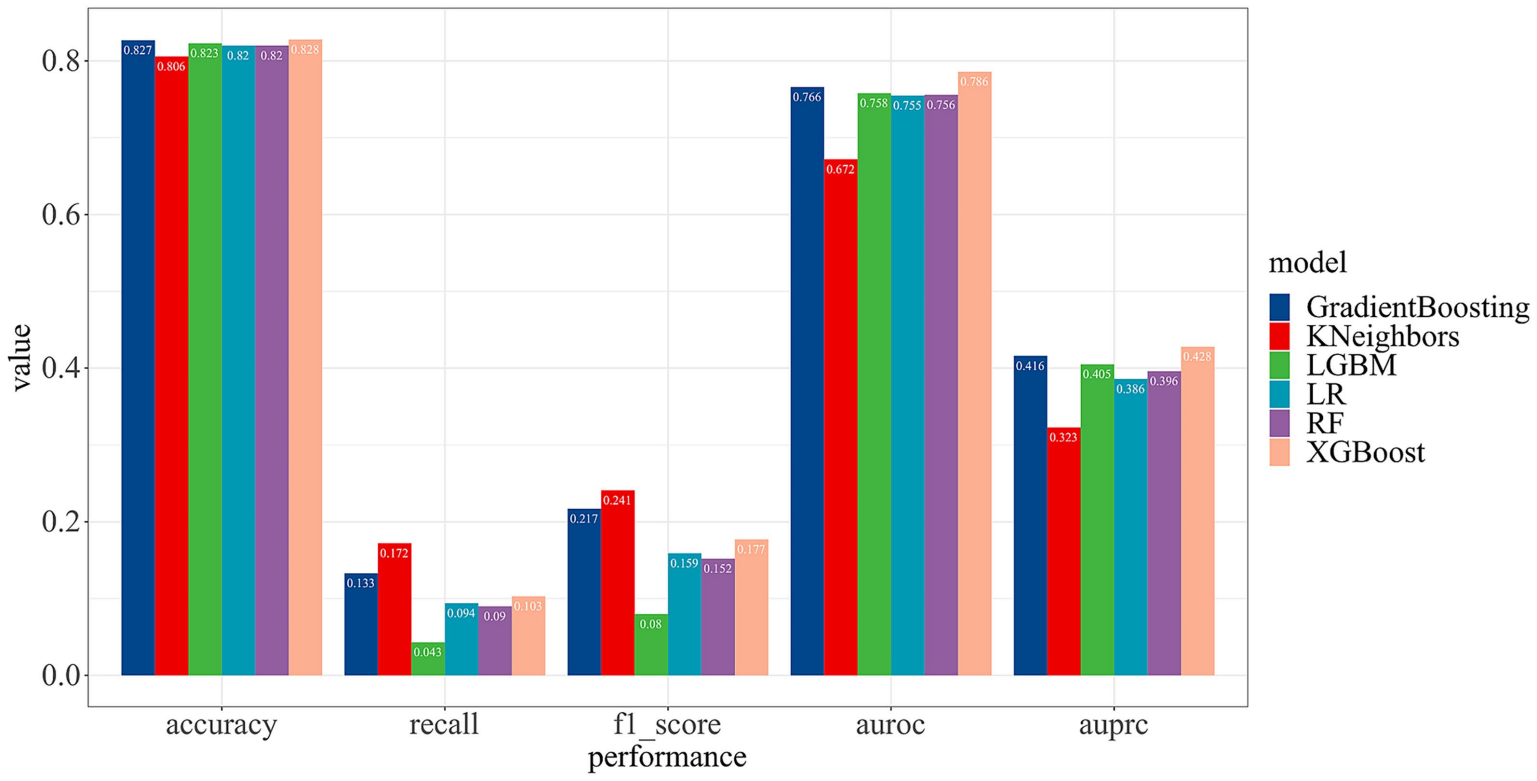
The performance comparison of different machine learning models.

To optimize the predictive capabilities of our model, we employed six machine learning models for model development. A comparative analysis of their performance metrics revealed that the KNN model performed the least effectively. In contrast, the XGBoost model demonstrated superior performance compared to the other methods, achieving an accuracy of 82.82% and the area under the receiver operating characteristic (AUROC) of 78.60%. Detailed overview of the performance for each ML model is showed in Figure 4.

### 3.4 Discrimination and consistency

After comparing of various machine learning models, we selected the XGBoost model to construct a prediction model for SA among community-dwelling older adults. Furthermore, this model was subjected to evaluate its discrimination ability and consistency.

Discrimination ability, a pivotal metric for gauging the model’s predictive prowess, pertains to its capacity to differentiate between individuals exhibiting successful aging within the community. We evaluated the model’s discriminative ability and consistency through AUROC and discriminant slope, which are critical benchmarks for assessing the reliability of our predictive outcomes.

Consistency denotes the alignment between the model’s predictive probabilities and the actual occurrence probabilities, visualized through a violin diagram. Meanwhile, we utilized the Brier score and calibration plots to conduct an exhaustive analysis of the model’s consistency. To bolster the study’s objectivity and applicability, we performed an in-depth evaluation of discrimination and consistency using models built with RF, LR, and XGBoost.

**Figure 5:**
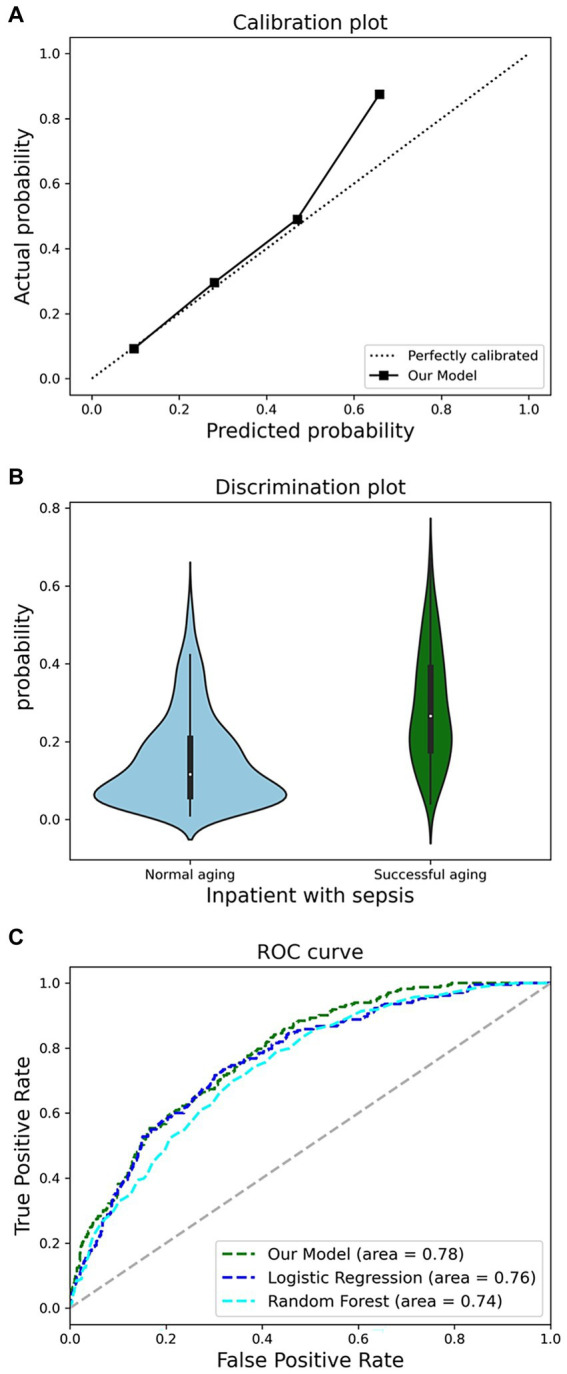
Comprehensive analysis of model performance metrics. **(A)**, Receiver operating characteristic (ROC) analysis. **(B)**, discrimination potentials of the prediction model in internal validation. **(C)**, Calibration potentials of the prediction model in internal validation.

Figure 5A illustrates the model’s AUROC, demonstrating superior performance of the XGBoost model compared to RF and LR. Our model achieved a discriminant slope of 0.140. The violin diagram indicates the model’s proficiency in accurately predicting true negatives while sustaining the risk distribution stability of true positives in Figure 5B. The calibration plot reveals a strong concordance between our model’s predictions and actual outcomes in Figure 5C. With a Brier score of 0.124, below the threshold of 0.25, our model demonstrates high predictive accuracy within the dataset.

### 3.5 Decision curve analysis

To assess the model’s performance in a manner that is aligned with clinical decision-making, we employed Decision Curve Analysis (DCA) to evaluate the “net benefit” of our model across a range of clinical scenarios. The concept of “net benefit” encompasses both the advantages and disadvantages associated with false positive and false negative outcomes.

**Figure 6:**
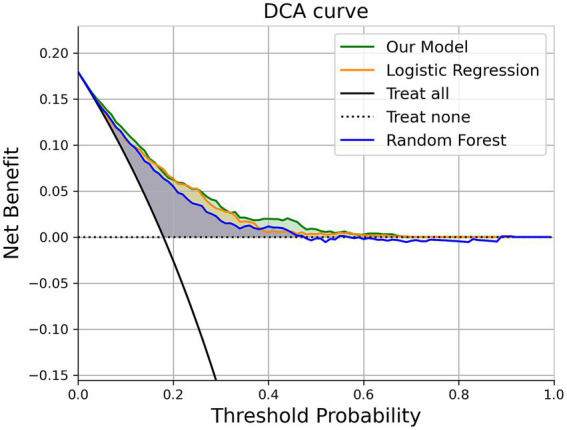
The decision curve analysis of the prediction model (XGBoost model).

DCA’s fundamental principle is to measure the prediction model’s utility at a specific clinical decision threshold. The prediction model in our study demonstrates an outstanding clinical net benefit in Figure 6.

### 3.6 Subgroup analysis

The model demonstrated varying performance across demographic subgroups in Table 4. Minimal disparity was observed, with nearly identical F1-scores (male: 0.484; female: 0.485). Performance declined in older adults (Age ≥70: F1 = 0.418, recall = 0.457) compared to younger counterparts (Age <70: F1 = 0.521, recall = 0.624), indicating higher false-negative rates. Significant income-based differences emerged, with the highest-income group achieving superior performance (F1 = 0.529) versus the lowest-income group (F1 = 0.318), which exhibited high false positives (precision = 0.182). Rural populations showed robust results (F1 = 0.597), while urban residents had notably lower recall (0.436), suggesting underdetection of positive cases.

**Table 4:** Subgroup performance and fairness analysis.

Subgroup	Recall	Specificity	Precision	F1-Score	AUROC	Key Misclassification Patterns
Gender (Male)	0.742	0.831	0.412	0.484	0.787	Balanced FP/FN across genders
Gender (Female)	0.748	0.829	0.410	0.485	0.789	/
Age (<70 years)	0.624	0.880	0.560	0.521	0.802	Low FN risk
Age (≥70 years)	0.457	0.901	0.385	0.418	0.752	High FN (missed SA cases)
Income (Low)	0.509	0.701	0.182	0.318	0.692	High FP (false alarms)
Income (High)	0.692	0.912	0.625	0.529	0.841	Low misclassification
Residence (Rural)	0.802	0.852	0.621	0.597	0.821	Robust performance
Residence (Urban)	0.436	0.885	0.320	0.370	0.731	High FN (missed SA cases)

FN, False Negative; FP, False Positives.

## 4 Discussion

In our study, we employed the representative cross-sectional data from the CHARLS 2020 to identify predictive factors of SA within Chinese community-dwelling older populations. By employing SHAP values and variables from Chinese community health records, we selected the predictive factors. Subsequently, we engaged six different machine learning models to construct a prediction model. We assessed the differentiation, consistency, and clinical value of the model. Our efforts culminated in the development of a SA prediction model that not only possesses strong interpretability, but also promises ease of application and broad utility within community settings. Finally, a prediction model of SA among Chinese community-dwelling older population was constructed with strong interpretation, easy application and promotion.

Firstly, we employed Rowe and Kahn’s widely recognized model of SA, encompassing physiological, psychological, cognitive, and social dimensions. Previous research has utilized various machine learning models to construct SA prediction models, yet the lack of a unified definition of SA, leading to model heterogeneity and limited comparability (38, 39). Our approach aims to address this by leveraging a comprehensive dataset to enhance model generalizability and applicability.

Asghari Varzaneh et al. (38) utilized single-factor regression on Iranian older population cross-sectional data to identify characteristic variables, ultimately developing a KNN-based ensemble model with a 93% accuracy and 96.10% AUC, demonstrating robust performance and discrimination. Similarly, Yazdani et al. (39) conducted a retrospective analysis on Iranian older individuals, employing principal component analysis to extract features, and developed an ANFIS model based on fuzzy C-means, integrating neural networks and fuzzy reasoning systems, which showed promising capabilities. However, both studies omitted the psychological dimension of SA, and their single-center, retrospective nature limits the assessment of model predictive performance in community-dwelling older populations.

In addition, Cai et al. (40) developed four SA prediction models based on physical fitness tests using 3-year follow-up data from 3,657 older individuals in Nanchang, with the deep learning model exhibiting the best predictive performance (AUC 90.0%). However, the reliance on physical fitness tests presents practical challenges for using in community, given the resource-intensive nature of such assessments.

Through literature review, this study pioneers the use of a representative and high-confidence Chinese database to construct an SA prediction model for community-dwelling older populations, offering a valuable tool for health assessment in Chinese communities. Our focus on community application aligns with previous suggestions that ML models can directly serve clinical practice, with the potential to provide SA prediction services, which are crucial for proactively addressing population aging. By embedding prediction models within electronic medical records and extending their reach to mobile applications, we enhance their utility in community settings (41).

In variable selection, we prioritized clinically available variables, improving the model’s generalizability in community primary care. Community primary care physicians establish health records, assess the older adult’s health status and quality of life, and identify modifiable health factors, enabling more targeted health care and disease management recommendation (42, 43). Our approach to identifying SA predictors based on health ecology aids in understanding the multi-dimensional influencing factors of individual SA in community-dwelling older populations (44).

To bridge the gap between model development and clinical application, we have incorporated insights from recent studies on ML deployment in primary care. These works underscore critical challenges—data integration (e.g., interoperability between EHRs and predictive tools), scalability (e.g., adapting models to diverse clinic workflows), and clinician acceptance (e.g., addressing “black-box” skepticism through interpretability features)—which directly informed our implementation strategy. The introduction of the SHAP method in our study enhances model interpretability by assessing the importance and interrelationships of different variables, aligning the new model’s discriminant pathways and clinical variable thresholds with current community clinical practices (45, 46). Our DCA indicates the model’s good clinical benefits, suggesting its importance in primary care scenarios.

However, our study is not without limitations. The lack of a unified concept and measurement method for SA may introduce population bias due to regional and usage scenario differences. The database’s limitations precluded the inclusion of potential key variables, and as a retrospective study, data missing and input errors are inevitable. While our study improves model practicality through health ecology and initial screening of characteristic variables in primary care health records, potential differences in performance and discrimination across various primary care scenarios may exist, necessitating prospective validation in future research.

Our subgroup analysis revealed important disparities in model performance across demographic groups, with significant implications for clinical implementation. While demonstrating gender fairness, the model showed reduced accuracy among older adults (≥70 years; recall = 0.457), urban (recall = 0.436), and low-income populations (precision = 0.182), reflecting distinct error patterns with clinical consequences. The elevated false-positive rate in low-income groups risks unnecessary interventions, whereas the higher false-negative rates among older adults and urban residents could delay critical care - a particular concern given these populations’ healthcare access challenges. These findings underscore the necessity for subgroup-specific model calibration and highlight key ethical considerations for deploying predictive algorithms in real-world geriatric care settings, where equitable performance across vulnerable populations is paramount. Future iterations should prioritize feature engineering to address these identified gaps while maintaining the model’s overall predictive utility.

Future studies will track regular follow-up results from CHARLS, extract longitudinal data of older individuals in Chinese communities, further refine the SA prediction model, and deploy the model as an online resource, providing community physicians and geriatricians with a more accessible tool for older health assessment. Embedding SA predictions into routine health assessments (e.g., annual older population check-ups) via standardized application programming interfaces, with modifiable risk factors (e.g., smoking, physical inactivity) flagged for clinician review. Prospective validation will conduct for new patient data (e.g., third quarter of 2025), and indicators were adjusted according to real-world noise (for example, AUPRC for class imbalance). Subgroup-specific thresholds (e.g., lower probability cutoff for older adults) and feature engineering (e.g., neighborhood-level variables for urban residents) will reduce bias in future deployments. After the release of the follow-up CHARLS data in the future, we will actively refer to pioneering longitudinal studies (such as HRS in the United States and ELSA in the United Kingdom) and compare their methods, advantages, and limitations with ours to improve the cross-cultural and cross regional applicability of the model.

## 5 Conclusion

In this study, we developed a novel prediction model for successful aging in Chinese community-dwelling older adults using CHARLS 2020 data and various machine learning models, demonstrating outstanding predictive performance and clinical net benefits. The model is highly interpretable and generalizable, offering significant utility in community primary healthcare settings. It provides insights into the factors influencing successful aging and aids healthcare providers in making targeted interventions to develop the quality of life for the Chinese community-dwelling older adult.

## Data Availability

The original contributions presented in the study are included in the article/Supplementary material, further inquiries can be directed to the corresponding author.

## References

[1] Chen X Giles J Yao Y Yip W Meng Q Berkman L. The path to healthy ageing in China: a Peking University-lancet commission. Lancet (London, England). (2022) 400:1967–2006. doi: 10.1016/s0140-6736(22)01546-x, PMID: 36423650 PMC9801271

[2] National Health Commission NOoA. Communique on the development of the National Cause for aging in 2022. (2023). Available at: https://www.gov.cn/lianbo/bumen/202312/content_6920261.htm (Accessed September 1, 2025).

[3] Wang H Qin D Fang L Liu H Song P. Addressing healthy aging in China: practices and prospects. Biosci Trends. (2024) 18:212–8. doi: 10.5582/bst.2024.01180, PMID: 38987161

[4] Li X Lu J Hu S Cheng KK De Maeseneer J Meng Q. The primary health-care system in China. Lancet (London, England). (2017) 390:2584–94. doi: 10.1016/s0140-6736(17)33109-4, PMID: 29231837

[5] Zhang Q. Primary care and all-cause mortality in urban China: a mixed-level analysis. Fam Pract. (2021) 38:121–6. doi: 10.1093/fampra/cmaa095, PMID: 32918446

[6] Rowe JW Kahn RL. Human aging: usual and successful. Science (New York, NY). (1987) 237:143–9. doi: 10.1126/science.3299702, PMID: 3299702

[7] Havighurst RJ. Successful aging. Gerontologist. (1961) 1:8–13.

[8] Rowe JW Kahn RL. Successful Aging. The Gerontologist. (1997) 37:433–40. doi: 10.1093/geront/37.4.433, PMID: 9279031

[9] Nakagawa T Cho J Yeung DY. Successful aging in East Asia: comparison among China, Korea, and Japan. J Gerontol B Psychol Sci Soc Sci. (2021) 76:S17–26. doi: 10.1093/geronb/gbaa042, PMID: 32324214 PMC8495754

[10] Bronfenbrenner U. Ecological system theory. Ann Child Dev. (1989) 6:187–249.

[11] Venkatapuram S Amuthavalli Thiyagarajan J. The capability approach and the WHO healthy ageing framework (for the un decade of healthy ageing). Age Ageing. (2023) 52:iv6–9. doi: 10.1093/ageing/afad126, PMID: 37902511 PMC10615052

[12] Rudnicka E Napierała P Podfigurna A Męczekalski B Smolarczyk R Grymowicz M. The World Health Organization (who) approach to healthy ageing. Maturitas. (2020) 139:6–11. doi: 10.1016/j.maturitas.2020.05.018, PMID: 32747042 PMC7250103

[13] Freund AM Baltes PB. Selection, optimization, and compensation as strategies of life management: correlations with subjective indicators of successful aging. Psychol Aging. (1998) 13:531–43. doi: 10.1037//0882-7974.13.4.531, PMID: 9883454

[14] Manierre M. Successful present, successful future? Assessment of a nonbinary model of successful aging. The Gerontologist. (2019) 59:727–37. doi: 10.1093/geront/gnx198, PMID: 29309575

[15] Cosco TD Stephan BC Brayne C. Validation of an a priori, index model of successful aging in a population-based cohort study: the successful aging index. Int Psychogeriatr. (2015) 27:1971–7. doi: 10.1017/s1041610215000708, PMID: 25990513

[16] Depp CA Jeste DV. Definitions and predictors of successful aging: a comprehensive review of larger quantitative studies. Am J Geriatr Psychiatry. (2006) 14:6–20. doi: 10.1097/01.JGP.0000192501.03069.bc, PMID: 16407577

[17] Zhao Y Hu Y Smith JP Strauss J Yang G. Cohort profile: the China health and retirement longitudinal study (Charls). Int J Epidemiol. (2014) 43:61–8. doi: 10.1093/ije/dys203, PMID: 23243115 PMC3937970

[18] Zhao Y Strauss J Chen X Wang Y Gong J Meng Q. China health and retire Ment longitudinal study wave 4 user’s guide National School of development, Peking University (2020). Available at: https://charls.pku.edu.cn/en/data/User2018.pdf (Accessed September 1, 2025).

[19] Dixon A. The United Nations decade of healthy ageing requires concerted global action. Nat Aging. (2021) 1:2. doi: 10.1038/s43587-020-00011-5, PMID: 37118001

[20] Nagarajan NR Teixeira AAC Silva ST. Ageing population: identifying the determinants of ageing in the least developed countries. Popul Res Policy Rev. (2021) 40:187–210. doi: 10.1007/s11113-020-09571-1

[21] Lin K Ning Y Mumtaz A Li H. Exploring the relationships between four aging ideals: a bibliometric study. Front Public Health. (2021) 9:762591. doi: 10.3389/fpubh.2021.762591, PMID: 35127615 PMC8814111

[22] Tian L Ding P Kuang X Ai W Shi H. The association between sleep duration trajectories and successful aging: a population-based cohort study. BMC Public Health. (2024) 24:3029. doi: 10.1186/s12889-024-20524-7, PMID: 39482676 PMC11529308

[23] ZhuXZhangXDingLTangYXuAYangF. Associations of pain and sarcopenia with successful aging among older people in China: evidence from Charls. J Nutr Health Aging. (2023) 27:196–201. doi: 10.1007/s12603-023-1892-2, PMID: 36973927

[24] Zheng A Casari A. Feature engineering for machine learning: principles and techniques for data scientists. Sevastopol, California: O'Reilly Media, Inc. (2018).

[25] McLeroy KR Bibeau D Steckler A Glanz K. An ecological perspective on health promotion programs. Health Educ Q. (1988) 15:351–77. doi: 10.1177/109019818801500401, PMID: 3068205

[26] Chang H Zhou J Wang Z. Multidimensional factors affecting successful aging among empty-nesters in China based on social-ecological system theory. Int J Environ Res Public Health. (2022) 19:11885. doi: 10.3390/ijerph191911885, PMID: 36231187 PMC9565406

[27] Anton SD Woods AJ Ashizawa T Barb D Buford TW Carter CS. Successful aging: advancing the science of physical Independence in older adults. Ageing Res Rev. (2015) 24:304–27. doi: 10.1016/j.arr.2015.09.005, PMID: 26462882 PMC4661112

[28] Rodrigues CE Grandt CL Alwafa RA Badrasawi M Aleksandrova K. Determinants and indicators of successful aging as a multidimensional outcome: a systematic review of longitudinal studies. Front Public Health. (2023) 11:1258280. doi: 10.3389/fpubh.2023.1258280, PMID: 38074742 PMC10703300

[29] Meurer WJ Tolles J. Logistic regression diagnostics: understanding how well a model predicts outcomes. JAMA. (2017) 317:1068–9. doi: 10.1001/jama.2016.20441, PMID: 28291878

[30] Cutler A Cutler DR Stevens JR. Random forests. In: Zhang C Ma Y, editors. Ensemble machine learning: methods and applications. New York, NY: Springer New York (2012). p. 157–75.

[31] Biau G. Analysis of a random forests model. J Mach Learn Res. (2012) 13:1063–95. doi: 10.1109/tase.2012.2183739

[32] Ke GL Meng Q Finley T Wang TF Chen W Ma WD Lightgbm: a highly efficient gradient boosting decision tree. Advances in neural information processing systems 30 (NIPS 2017) (2017).

[33] Zuo D Yang L Jin Y Qi H Liu Y Ren L. Machine learning-based models for the prediction of breast Cancer recurrence risk. BMC Med Inform Decis Mak. (2023) 23:276. doi: 10.1186/s12911-023-02377-z, PMID: 38031071 PMC10688055

[34] Yuan JD Douzal-Chouakria A Yazdi SV Wang ZH. A large margin time series nearest neighbour classification under locally weighted time warps. Knowl Inf Syst. (2019) 59:117–35. doi: 10.1007/s10115-018-1184-z

[35] Chen T. Q. Guestrin C. Assoc Comp M. XGBoost: a scalable tree boosting system. Kdd'16: Proceedings of the 22nd ACM sigkdd international conference on knowledge discovery and data mining (2016). p. 785–94.

[36] Van Calster B Wynants L Verbeek JFM Verbakel JY Christodoulou E Vickers AJ. Reporting and interpreting decision curve analysis: a guide for investigators. Eur Urol. (2018) 74:796–804. doi: 10.1016/j.eururo.2018.08.038, PMID: 30241973 PMC6261531

[37] Su PY Wei YC Luo H Liu CH Huang WY Chen KF. Machine learning models for predicting influential factors of early outcomes in acute ischemic stroke: registry-based study. JMIR Med Inform. (2022) 10:e32508. doi: 10.2196/32508, PMID: 35072631 PMC8994144

[38] Asghari Varzaneh Z Shanbehzadeh M Kazemi-Arpanahi H. Prediction of successful aging using ensemble machine learning algorithms. BMC Med Inform Decis Mak. (2022) 22:258. doi: 10.1186/s12911-022-02001-6, PMID: 36192713 PMC9527392

[39] Yazdani A Shanbehzadeh M Kazemi-Arpanahi H. Using an adaptive network-based fuzzy inference system for prediction of successful aging: a comparison with common machine learning algorithms. BMC Med Inform Decis Mak. (2023) 23:229. doi: 10.1186/s12911-023-02335-9, PMID: 37858200 PMC10585757

[40] Cai T Long J Kuang J You F Zou T Wu L. Applying machine learning methods to develop a successful aging maintenance prediction model based on physical fitness tests. Geriatr Gerontol Int. (2020) 20:637–42. doi: 10.1111/ggi.13926, PMID: 32358851

[41] Brothers A Kornadt AE Nehrkorn-Bailey A Wahl HW Diehl M. The effects of age stereotypes on physical and mental health are mediated by self-perceptions of aging. J Gerontol B Phychol Sci Soc Sci. (2021) 76:845–57. doi: 10.1093/geronb/gbaa176, PMID: 33057726 PMC8063677

[42] Georgiev K Wang Y Conkie A Sinclair A Christodoulou V Seyedzadeh S. Predicting incident dementia in community-dwelling older adults using primary and secondary care data from electronic health records. Brain Commun. (2025) 7:fcae469. doi: 10.1093/braincomms/fcae469, PMID: 39759471 PMC11697165

[43] Nguyen OT Kunta AR Katoju S Gheytasvand S Masoumi N Tavasolian R. Electronic health record nudges and health care quality and outcomes in primary care: a systematic review. JAMA Netw Open. (2024) 7:e2432760. doi: 10.1001/jamanetworkopen.2024.32760, PMID: 39287947 PMC11409160

[44] Ko PC Yeung WJ. An ecological framework for active aging in China. J Aging Health. (2018) 30:1642–76. doi: 10.1177/0898264318795564, PMID: 30160571

[45] Niu T Cao S Cheng J Zhang Y Zhang Z Xue R. An explainable predictive model for anxiety symptoms risk among Chinese older adults with abdominal obesity using a machine learning and Shapley additive explanations approach. Front Psych. (2024) 15:1451703. doi: 10.3389/fpsyt.2024.1451703, PMID: 39720434 PMC11666561

[46] Han Y Wang S. Disability risk prediction model based on machine learning among Chinese healthy older adults: results from the China health and retirement longitudinal study. Front Public Health. (2023) 11:1271595. doi: 10.3389/fpubh.2023.1271595, PMID: 38026309 PMC10665855

