# Supplementary material for: Development and validation of a successful aging prediction model for older adults in China based on health ecology theory
Source: Front Public Health. 2025 Oct 13;13:1595540. doi: 10.3389/fpubh.2025.1595540 (PMC12554565; doi:10.3389/fpubh.2025.1595540)
Supplement: Supplementary file 2 [file Table_1.DOCX]

Supplement Table 1. Subgroup Performance and Key Findings.

| Subgroup | Performance Metrics | Key Findings |
| --- | --- | --- |
| Gender | Male (F1=0.484) vs. Female (F1=0.485) | Minimal bias: Similar precision/recall balance across genders. |
| Age | <70y (F1=0.521) vs. ≥70y (F1=0.418) | Age bias: Lower recall (0.457) in older adults due to complex comorbidities. |
| Income | High-income (F1=0.529) vs. Low-income (F1=0.318) | Income bias: High FP rate in low-income groups (precision=0.182). |
| Residence | Urban (F1=0.370) vs. Rural (F1=0.597) | Urban bias: Low recall (0.436) in urban areas due to heterogeneous lifestyles. |

Notes: Abbreviation: FP = False Positives.

Supplement Table 2. Classification metrics.

| Metric | Value | Clinical Implication |
| --- | --- | --- |
| Sensitivity | 0.74 | Captures 74% of true SA cases |
| Specificity | 0.83 | Correctly rules out 83% of non-SA cases |
| Precision | 0.41 | 41% of predicted SA cases are correct |
| F1-Score | 0.52 | Balance between recall and precision |
